# *Smad4* restricts injury-provoked biliary proliferation and carcinogenesis

**DOI:** 10.1242/dmm.050358

**Published:** 2024-02-28

**Authors:** William B. Alexander, Wenjia Wang, Margaret A. Hill, Michael R. O'Dell, Luis I. Ruffolo, Bing Guo, Katherine M. Jackson, Nicholas Ullman, Scott C. Friedland, Matthew N. McCall, Ankit Patel, Nathania Figueroa-Guilliani, Mary Georger, Brian A. Belt, Christa L. Whitney-Miller, David C. Linehan, Patrick J. Murphy, Aram F. Hezel

**Affiliations:** ^1^ Department of Biomedical Genetics, University of Rochester Medical Center, Rochester, NY 14642, USA; ^2^ Department of Medicine, Hematology/Oncology, Wilmot Cancer Institute, University of Rochester Medical Center, Rochester, NY 14642, USA; ^3^ Department of Surgery, University of Rochester Medical Center, Rochester, NY 14642, USA; ^4^ Department of Biostatistics and Computational Biology, University of Rochester Medical Center, Rochester, NY 14642, USA; ^5^ Department of Pathology and Laboratory Medicine, University of Rochester Medical Center, Rochester, NY 14642, USA

**Keywords:** Cholangiocarcinoma, Biliary epithelium, Murine models of liver injury, TGFβ/SMAD4, Methylation

## Abstract

Cholangiocarcinoma (CCA) is a deadly and heterogeneous type of cancer characterized by a spectrum of epidemiologic associations as well as genetic and epigenetic alterations. We seek to understand how these features inter-relate in the earliest phase of cancer development and through the course of disease progression. For this, we studied murine models of liver injury integrating the most commonly occurring gene mutations of CCA – including *Kras*, *Tp53*, *Arid1a* and *Smad4* – as well as murine hepatobiliary cancer models and derived primary cell lines based on these mutations. Among commonly mutated genes in CCA, we found that *Smad4* functions uniquely to restrict reactive cholangiocyte expansion to liver injury through restraint of the proliferative response. Inactivation of *Smad4* accelerates carcinogenesis, provoking pre-neoplastic biliary lesions and CCA development in an injury setting. Expression analyses of *Smad4*-perturbed reactive cholangiocytes and CCA lines demonstrated shared enriched pathways, including cell-cycle regulation, MYC signaling and oxidative phosphorylation, suggesting that *Smad4* may act via these mechanisms to regulate cholangiocyte proliferation and progression to CCA. Overall, we showed that TGFβ/SMAD4 signaling serves as a critical barrier restraining cholangiocyte expansion and malignant transformation in states of biliary injury.

## Teaser

**Summary:** *Smad4*, a commonly mutated gene in cholangiocarcinoma, restricts the cholangiocyte proliferative response to liver injury and oncogenic transformation via known/novel transcriptomic changes and genomic methylation patterns.

**Research Simplified**

Cholangiocarcinoma, the cancer of bile ducts in the liver, is one of the deadliest cancers. This is mostly because it is often only diagnosed once the disease has already spread throughout the body; it also responds poorly to treatment. Gaining a better understanding of how the cancer develops can lead to earlier diagnosis and improved therapies that will save patients' lives.

To better understand the cause of cholangiocarcinoma, the authors of this study studied the relationship between the inactivation of several genes and injury to the liver. Among these genes, SMAD4 stood out. This gene encodes a protein that transmits signals in the cell to its nucleus to coordinate a response that prevents cells from growing too fast. This means that inactivation of SMAD4 will result in overgrowth of cells in the liver. The authors found that when inactivation of SMAD4 is combined with liver injury, it causes the tissue to become cancerous.

This study offers an important piece in the puzzle of what causes cholangiocarcinoma. Investigating this further could help doctors diagnose cholangiocarcinoma earlier and help researchers develop effective treatments to reduce the devastating impact of this rare cancer.

## INTRODUCTION

Cholangiocarcinoma (CCA) is a lethal malignancy due to its resistance to chemotherapies and its frequent late-stage diagnosis. Risk factors for CCA include conditions associated with biliary tract injury, including primary sclerosing cholangitis, hepatolithiasis and infection by liver flukes, as well as conditions associated with hepatocellular injury, cirrhosis and viral hepatitis (Banales et al., 2016; Llovet et al., 2016). The mutational spectrum of CCA includes mutations in *TP53*, *KRAS*, *IDH1* and *IDH2*, *SMAD4*, and *ARID1A* (Chan-On et al., 2013; Jusakul et al., 2017; Nakamura et al., 2015). Several studies have identified distinct mutational signatures related to the disease etiology, including enrichment of *SMAD4* and *TP53* mutations in subsets of CCA associated with infection by liver flukes. These observations highlight how inciting risk factors may influence tumor evolution and suggest that some mutations may be uniquely advantageous to tumor development in such settings.

SMAD4 functions as the central downstream mediator of the TGFβ superfamily, a class of related proteins including TGFβs and BMPs, which play critical roles in development, tissue homeostasis and cancer (Ikushima and Miyazono, 2010; Wakefield and Hill, 2013; Wu and Hill, 2009). Genetic disruption of the TGFβ/SMAD4-signaling axis cooperates with *Pten* deletion to promote hepatic oncogenesis; however, *Pten* mutations are uncommon in CCA and even less frequently associated with mutations in TGFβ/SMAD4 components (Mu et al., 2016; Xu et al., 2006). How *SMAD4* interacts with common mutations found in human CCA, and what role injury plays in this process, are unknown. In the present study, we established a unique role for *Smad4* in suppressing biliary proliferation in response to injury and linked this to its action as a tumor suppressor in multiple autochthonous mouse models based on *Kras* and *Tp53* mutations. Finally, we demonstrated that *Smad4* may function at the epigenetic level to regulate genomic methylation patterns.

## RESULTS

### *Smad4* uniquely suppresses cholangiocyte proliferation in response to injury

A hallmark response of the liver to a variety of acute and chronic injuries is the ductular reaction, recognized as a proliferative expansion of cholangiocytes around the portal triad (Jors et al., 2015; Stanger, 2015). Moreover, these reactions are frequently identified in human tissue in association with CCA and are the earliest events found in genetically engineered mouse models of the disease (Endo et al., 2002; Saha et al., 2014). These observations suggest ductular reactions represent a premalignant state that is susceptible to transformation when affected by additional oncogenic hits (Zhu et al., 2016).

**Fig. 1.**
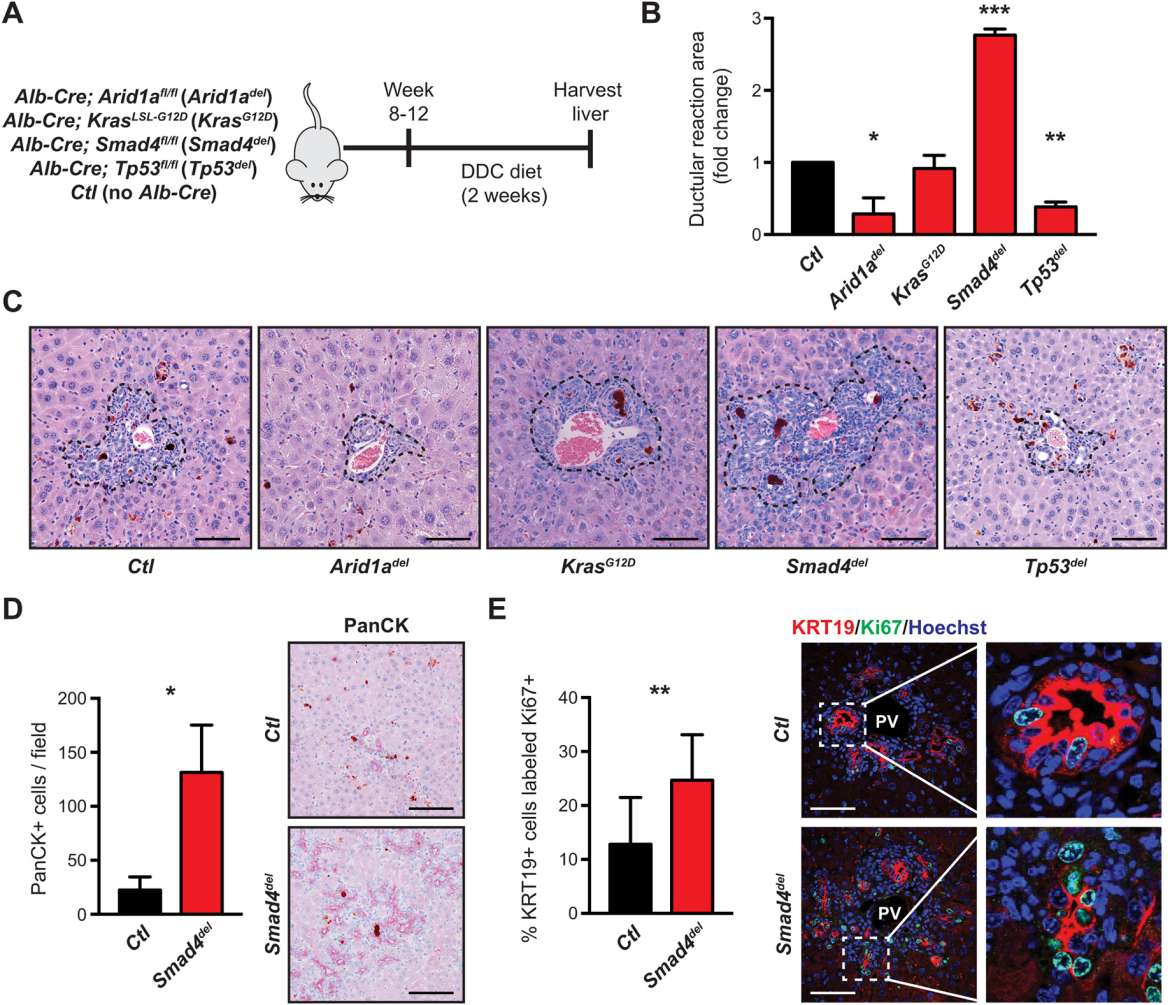
***Smad4* suppresses cholangiocyte proliferation in response to injury.** (A) Generation of compound mutant mice carrying *Alb-Cre* and *Arid1a^fl/fl^* (*Arid1a^del^*), *Kras^LSL-G12D^* (*Kras^G12D^*), *Smad4^fl/fl^* (*Smad4^del^*) or *Tp53^fl/fl^* (*Tp53^del^*). Experimental and control cohorts [no *Alb-Cre*, (*Ctl*)] were put on a DDC diet for 2 weeks and their livers were harvested, *n*=3-4 mice per cohort. (B) Quantification of the ductular reaction area of the experimental cohorts compared to the control cohort as outlined in A, showing *Smad4^del^* with a significant expansion of the biliary compartment. (C) Representative images of ductular reactions (surrounded by dashed lines), magnified 200×. (D) Quantification of reactive cholangiocytes (identified as PanCK+) in *Smad4^del^* and *Ctl* livers demonstrating more reactive cholangiocytes in the DDC-injured *Smad4del* livers compared to that of *Ctl*. Three random images per mouse (magnified 100×) were used for quantification. Representative ductular reactions are shown on the right. (E) Quantification of proliferating cholangiocytes (KRT19+ and Ki67+) in *Smad4^del^* and *Ctl* livers revealing more proliferative cholangiocytes in DDC-injured *Smad4del* livers compared to that of *Ctl*. For quantification, three random portal fields per mouse were aggregated by genotype. Representative images (magnified 100×) are shown; boxed areas are shown enlarged on the right. *P<0.05, **P<0.01, ***P<0.0001; scale bars: 50 µm (C) and 100 µm (D,E).

Given biliary injury is a risk factor for CCA, we evaluated how commonly mutated genes in CCA, including *SMAD4*, *TP53*, *ARID1A* and the proto-oncogene *KRAS*, impact the ductular reaction. The *Alb-Cre* allele was employed to generate liver specific mutations – deletion of *Tp53*, *Smad4* or *Arid1a*, as well as expression of constitutively active *Kras^G12D^* (*n*=3-4 mice per cohort) – followed by 2 weeks of dietary 3,5-diethoxycarbonyl-1,4-dihydrocollidine (DDC) to provoke biliary injury through the accumulation of ‘porphyrin plugs’ (Fig. 1A) (Hill et al., 2018a). *Kras^G12D^* had no discernible impact on ductular reaction size, whereas *Arid1a* and *Tp53* deletions modestly suppressed ductular reaction. *Smad4* deletion led to a striking expansion of the biliary compartment (Fig. 1A-C). Porphyrin plug burden was comparable between experimental and control livers (Fig. S1A). Mechanical injury with bile-duct ligation (BDL) demonstrated a similar ductular expansion in the *Smad4^del^* cohort, albeit with a wider range of responses, likely due to the variable effect of ligation (Fig. S1B). Consistent with TGFβ pathway engagement in the injured liver, DDC-injured livers demonstrated increased expression of TGFβ ligands, and increased cytoplasmic and nuclear staining for phosphorylated (phospho)-SMAD2, indicating pathway activation (Fig. S1C,D). Thus, among the most frequently mutated genes in CCA, *Smad4* has a unique role in suppressing injury-induced ductular reaction.

The ductular reaction consists of a proliferative expansion of reactive cholangiocytes accompanied by fibrogenic and immune responses. While hepatocytes may contribute to the cholangiocyte population in certain contexts, pre-existing cholangiocytes are the principal cellular source of reactive cholangiocytes in the liver (Jors et al., 2015; Schaub et al., 2018; Yanger et al., 2013). Consistent with this, DDC-injured *Smad4^del^* livers harbored more reactive cholangiocytes (PanCK+) than controls (*Ctl*) (Fig. 1D) and these were also more proliferative (KRT19+/Ki67+) (Fig. 1E). Digestion and flow sorting of livers following control (*n*=3/cohort) or DDC diet (*n*=6/cohort) for general leukocytes revealed no significant difference between *Ctl* and *Smad4^del^* livers (Fig. S2A). We further investigated if *Smad4* deletion impacted recruitment of inflammatory monocytes/macrophages – the most common immune cells in ductular reactions and CCA and a promising therapeutic target – and, again, we found no significant difference of recruitment of monocytes/macrophages between *Ctl* and *Smad4^del^* livers following control diet or DDC diet (Fig. S2B) (Banales et al., 2016). Similarly, granulocyte recruitment was not impacted (Fig. S2B), and examination of the fibrogenic response, including myofibroblasts (αSMA+) and collagen content (staining with Sirius Red), showed no statistically significant differences in myofibroblast and collagen content (Fig. S2C,D). As *Smad4* has previously been implicated in the apoptotic response, the level of cleaved (i.e. activated) caspase 3 (CC3+) was evaluated, showing no significant difference between *Ctl* and *Smad4^del^* cohorts (Fig. S2E) (David et al., 2016).

### Gene set enrichment analysis of *Smad4*-perturbed reactive cholangiocytes implicates *Smad4* in proliferative, metabolic and inflammatory pathways

**Fig. 2.**
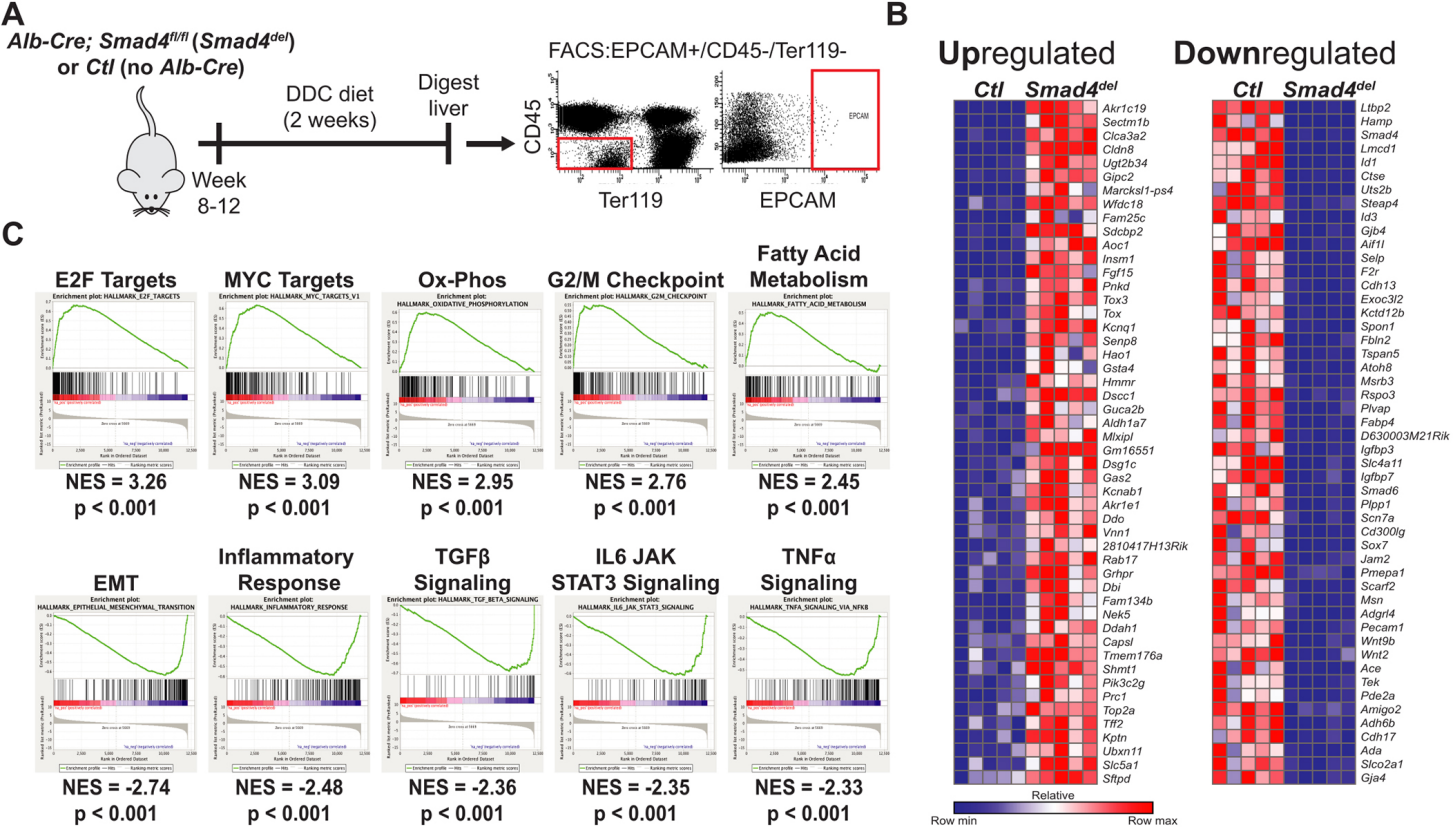
***Smad4* is involved in the regulation of pathways associated with proliferation, metabolism and inflammation.** (A) Schematic outlining the strategy to isolate and sequence reactive cholangiocytes. Livers are enzymatically digested and flow-sorted to isolate the EPCAM-positive/CD45-negative/Ter119-negative (EPCAM+/CD45-/Ter119-) population from which RNA had been prepared for RNA sequencing. Representative FACS plots are shown on the left. Boxed areas enclose cells of interest (P5 = CD45^−^/Ter119^−^ cells, EpCAM = EpCAM^+^ cells). *n*=5 per cohort. (B) Heat map showing the top 50 most significantly upregulated and downregulated genes in *Smad4^del^*-perturbed reactive cholangiocytes. (C) Gene set enrichment analysis (GSEA) of genes differentially expressed in *Smad4^del^*-perturbed reactive cholangiocytes. Enrichment plots from the Hallmarks Gene Set Collection are shown for the top five gene sets most positively correlated (top row) in genes upregulated in *Smad4^del^* cells or most negatively correlated (bottom row) in genes downregulated in *Smad4^del^* cells. Ox-Phos, oxidative phosphorylation; EMT, epithelial mesenchymal transition; NES, normalized enrichment score.

To understand the processes regulated by *Smad4* within reactive cholangiocytes, *Ctl* and *Smad4^del^* mice (*n*=5/cohort) were provided a DDC diet for 2 weeks (see Materials and Methods). Thereafter, reactive cholangiocytes were isolated from digested liver by using fluorescence-activated cell sorting (FACS), followed by RNA amplification and sequencing analysis (Fig. 2A) (Yanger et al., 2013; Yimlamai et al., 2014). Increased mRNA expression of the cholangiocyte markers *Krt19* and *Krt7* following this sorting process demonstrated its efficacy (Fig. S3). By further validating isolation of cholangiocytes within our dataset, we found the cholangiocyte markers *Spp1*, *Cyr61*, *Cdh1*, *Krt19* and *Epcam* to be among the top 100 most expressed genes within the entire transcriptome of both *Ctl* and *Smad4^del^* cohorts (pre-sort relative to post-sort). Differential expression analysis revealed that in *Smad4^del^*-perturbed reactive cholangiocytes were 939 significantly upregulated genes and 1337 significantly downregulated genes (adjusted *P*-value <0.05; Fig. 2B), including established *Smad4*-target genes, such as *Id1*, *Id3, Cdkn1a* and *Cdkn2b*. Gene set enrichment analysis (GSEA) of our list of differentially expressed genes revealed significant positive or negative enrichments (i.e. gene sets upregulated or downregulated, respectively, in *Smad4^del^*-perturbed reactive cholangiocytes) (Fig. 2C) (Liberzon et al., 2015; Subramanian et al., 2005). Positively correlated ‘E2F Targets’ and ‘G2/M Checkpoint’ gene sets were consistent with the proliferative phenotype in *Smad4^del^*-perturbed reactive cholangiocytes and the known functions of *Smad4* in controlling cell cycle progression. Negatively enriched gene sets included ‘TGFβ signaling’ and ‘Epithelial mesenchymal transition’, both of which are known to be regulated by *Smad4*, thus validating perturbation in our system. Pathways, such as oxidative phosphorylation and fatty acid metabolism, that have established importance in oncogenesis but are unknown to be governed by *Smad4*, were also identified.

### *Smad4* suppresses cancer development in the AKP and AP hepatobiliary cancer models

**Fig. 3.**
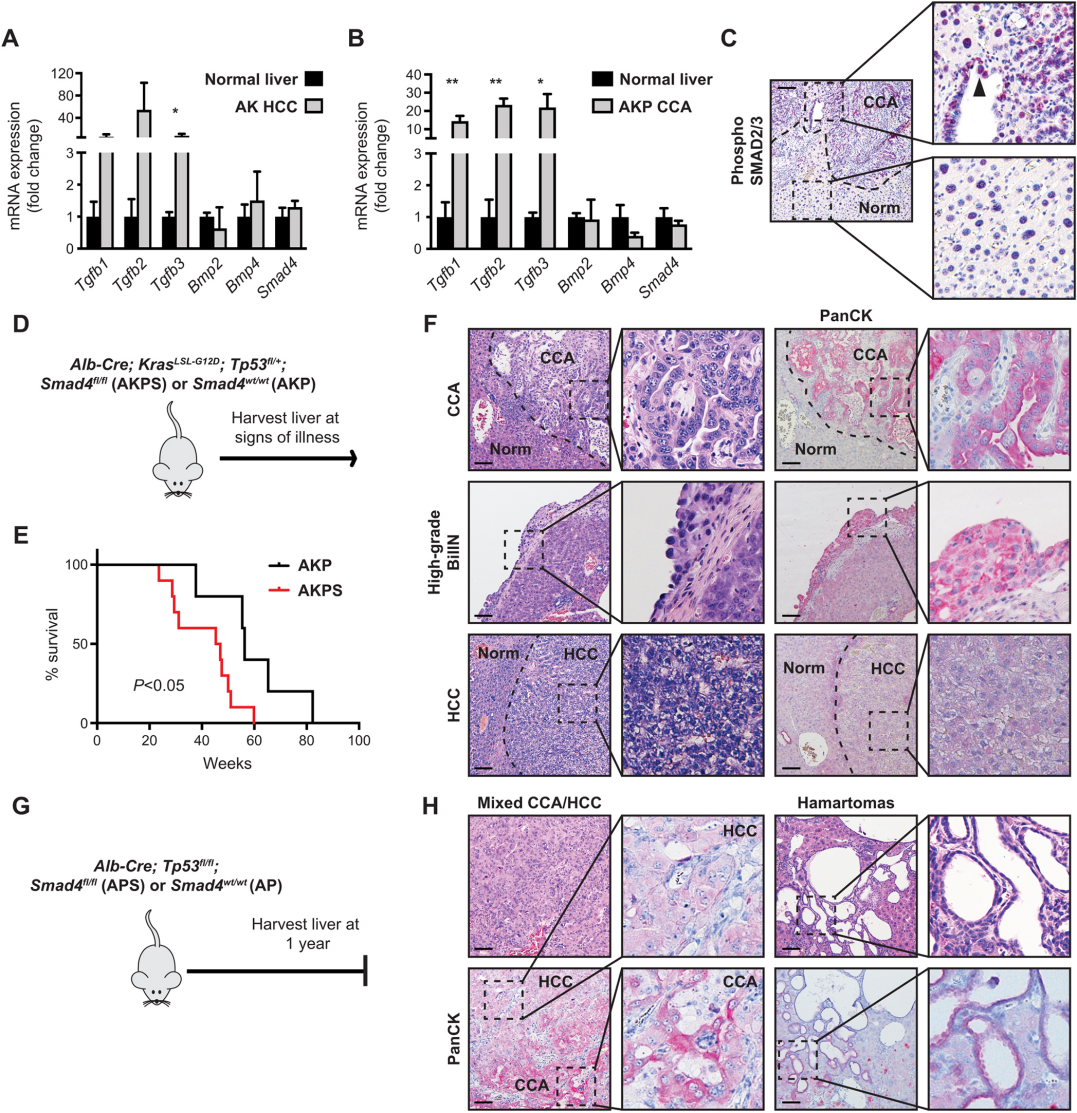
***Smad4* suppresses cancer progression in the AKP and AP hepatobiliary cancer models.** (A) mRNA expression levels assessed by qPCR of TGFβ family-related genes in (A) AK mouse model-derived HCC (AK HCC) or (B) AKP mouse model-derived CCA (AKP CCA) tissue compared to normal liver tissue showing increased expression of TGFβ ligands, indicating activation of the TGFβ pathway. Expression was normalized to *Rhoa*, *n*=3 mice per cohort. (C) Immunohistochemistry image of phosphorylated (phospho)-SMAD2 in AKP CCA tissue and adjacent normal (Norm) tissue (magnified 100×). Boxed areas are shown enlarged on the right, with CCA tissue at the top and normal tissue at the bottom. Arrowhead shows positive phospho-SMAD2 nuclei in malignant epithelium. (D) Compound mutant mice carrying *Alb-Cre*; *Kras^LSL-G12D^*; *Tp53^fl/+^* and either *Smad4^fl/fl^* or *Smad4^wt/wt^* alleles (AKPS or AKP mouse models, respectively) were followed for survival (*n*=5–10 mice per cohort). (E) Kaplan–Meier plot comparing survival curves of AKPS (red) and AKP (black) mice, showing decreased overall survival of the AKPS compared to the AKP cohort (*P*<0.05). (F) Representative histology and immunohistochemistry images of AKPS CCA, high-grade BilIN and HCC (magnified 100×). Boxed areas are shown enlarged to the right. Staining of reactive cholangiocytes (PanCK) highlights malignant biliary epithelium. (G) Compound mutant mice carrying *Alb-Cre*;*Tp53^fl/fl^* and *Smad4^fl/fl^* or *Smad4^wt/wt^* alleles (APS or AP mouse models, respectively) were followed and sacrificed at one year of age, *n*=8–15 mice per cohort. (H) Representative histology images of APS mixed CCA/HCC and hamartomas (magnified 100×). Boxed areas are shown enlarged to the right. Staining of PanCK highlights biliary differentiation. *P<0.05, **P<0.01, Scale bars: 50 µm.

Mutations in *SMAD4* are found coincidently with both *KRAS* and/or *TP53* mutations in humans (Nakamura et al., 2015). Thus, we investigated the impact of various combinations of mutations in mouse models (Chan-On et al., 2013; Jusakul et al., 2017). Expression of oncogenic *Kras^G12D^* driven by liver-specific Cre recombinase (*Alb-Cre*; *Kras*^*LSL-G12D*^, hereafter referred to as AK) leads to latent development of predominantly hepatocellular carcinoma (HCC) or mixed pathologies (O'Dell et al., 2012; Saha et al., 2014). This model exhibited activation of the TGFβ pathway, as indicated by increased expression of TGFβ ligands (Fig. 3A). We have previously established that targeting *Kras* and *Tp53* mutations to the mouse liver by using the *Alb-Cre* transgene (*Alb-Cre*; *Kras*^*LSL-G12D*^; *Tp53*^*fl/+*^, hereafter referred to as AKP) promotes development of CCA and, less frequently, a mix of CCA/HCC and pure HCC (Hill et al., 2018a,b; O'Dell et al., 2012). Similar to the AK model, we found elevated expression of TGFβ ligands in CCA derived from the AKP model; moreover, nuclear phospho-SMAD2 staining supported engagement of the TGFβ/SMAD4 tumor suppressor pathway, in accordance with other studies (Fig. 3B,C) (Mu et al., 2016). To further understand the function of *Smad4* in CCA, we targeted inactivating *Smad4* mutations along with *Kras* and *Tp*53 mutations driven by the *Alb-Cre* transgene (*Alb-Cre*; *Kras*^*LSL-G12D*^; *Tp*53^*fl/+*^; *Smad4*^*fl/fl*^, hereafter referred to as AKPS) (Fig. 3D). The survival and tumor spectrum results of the control AKP cohort were similar to those determined in our previously published study (Fig. S4A; Table S1; Fig. 3E) (O'Dell et al., 2012). The AKPS cohort demonstrated accelerated tumor development relative to the control cohort, with livers from all animals displaying multiple nodules with features of both CCA and HCC on pathology review, and as verified by cytokeratin staining (CCA marker) and arginase I (HCC marker) (Fig. 3D-F; Fig. S4A,B; Table S1). Precursor CCA lesions, including biliary intraepithelial neoplasia (BilIN) (Fig. 3F) and extensive biliary ductular reactions, were observed in four of nine mice (Table S1).

We also deleted *Smad4* in the context of *Tp53* deletion alone. A cohort of *Alb-Cre*; *Tp53^fl/fl^*; *Smad4^fl/fl^* (hereafter referred to as APS) mice was observed for 1 year, after which no mice exhibited signs of illness. Livers of APS and *Alb-Cre*; *Tp53^fl/fl^* (hereafter referred to as AP) mice were then harvested for histology analyses, showing mixed HCC/CCA tumors in ∼25% of APS mice but none of AP mice (Fig. 3G,H; Fig. S4C,D; Table S2). Additionally, ∼60% of the APS cohort exhibited PanCK+ cystic structures that are consistent with biliary hamartomas, and gross biliary cysts; however, no tumors or biliary lesions were observed in the AP control cohort (Fig. 3H; Fig. S4C,D). Together, these data demonstrate that *Smad4* suppresses both CCA and HCC development, and that biliary injury can further provoke premalignant biliary lesions and CCA development within a *Smad4*-null setting.

### Pathway analyses suggest *Smad4* has conserved functions in CCA lines and in reactive cholangiocytes

**Fig. 4.**
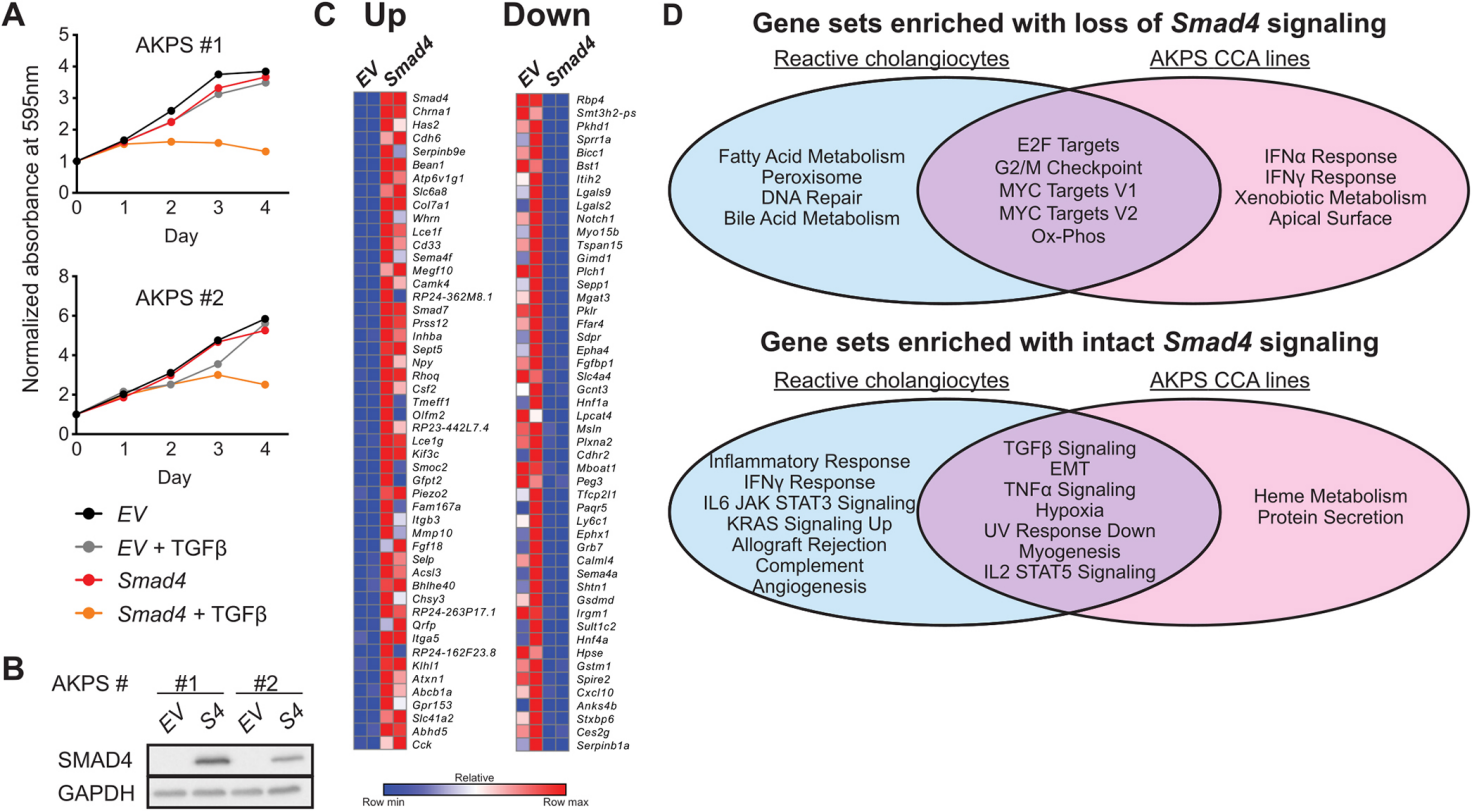
**Pathway analyses, suggesting conserved functions of *Smad4* in CCA and reactive cholangiocytes.** (A) Plotted are the growth curves of two CCA cell lines (#1 and #2) derived from liver tumors from mice in the AKPS cohort expressing only *Smad4* (red) or empty vector (*EV*; black), and *Smad4* or *EV* plus treatment with TGFβ (gray or orange, respectively), showing growth arrest/inhibition with TGFβ treatment following reintroduction of *Smad4* to both AKPS #1 and #2. (B) Western blot confirming restoration of *Smad4 (S4)* protein levels in the two AKPS lines assessed in A. (C) Heat map showing the top 50 most significantly up- and downregulated genes in *Smad4*-restored CCA cell lines. (D) Individual and shared gene sets enriched with loss of *Smad4* signaling (top) and with intact *Smad4* signaling (bottom) in AKPS CCA cell lines (right) compared to those enriched in reactive cholangiocytes (left).

Primary cancer cell lines derived from liver tumors from mice in the AKPS cohort demonstrated loss of SMAD4, expression of biliary markers, and absence of growth arrest/inhibition with TGFβ treatment (Fig. 4A,B; Fig. S5A). Reintroduction of *Smad4* through lentiviral transduction, confirmed by RT-PCR and western blot, led to TGFβ dependent growth inhibition (Fig. 4A,B). RNA-sequencing of TGFβ treated empty vector (*EV*) or *Smad4*-expressing AKPS cell lines (Fig. 4C) followed by GSEA revealed a number of gene sets overlapping with those obtained from analyses in reactive cholangiocytes. The hallmark gene sets MYC targets, E2F targets, G2/M checkpoint and oxidative phosphorylation were enriched with loss of *Smad4*, while – among others – TGFβ signaling, epithelial-to-mesenchymal transition (EMT) and TNFα signaling were enriched with intact *Smad4* signaling, suggesting conserved growth-suppressive functions of *Smad4* in primary cholangiocytes and advanced cancer (Fig. 4D; Fig. S5B). In accordance, preliminary evaluation of the Myc pathway in an AKPS cell line showed downregulation of several Myc target genes upon reintroduction of *Smad4*. However, interrogation of oxidative phosphorylation-related genes selected from GSEA-leading edge analysis did not show consistent dysregulation upon *Smad4* perturbation in this line (Fig. S5C); thus, if and how *Smad4* impacts oxidative phosphorylation in injury and CCA development remains to be further elucidated.

### *Smad4* is a determinant of DNA methylation

**Fig. 5.**
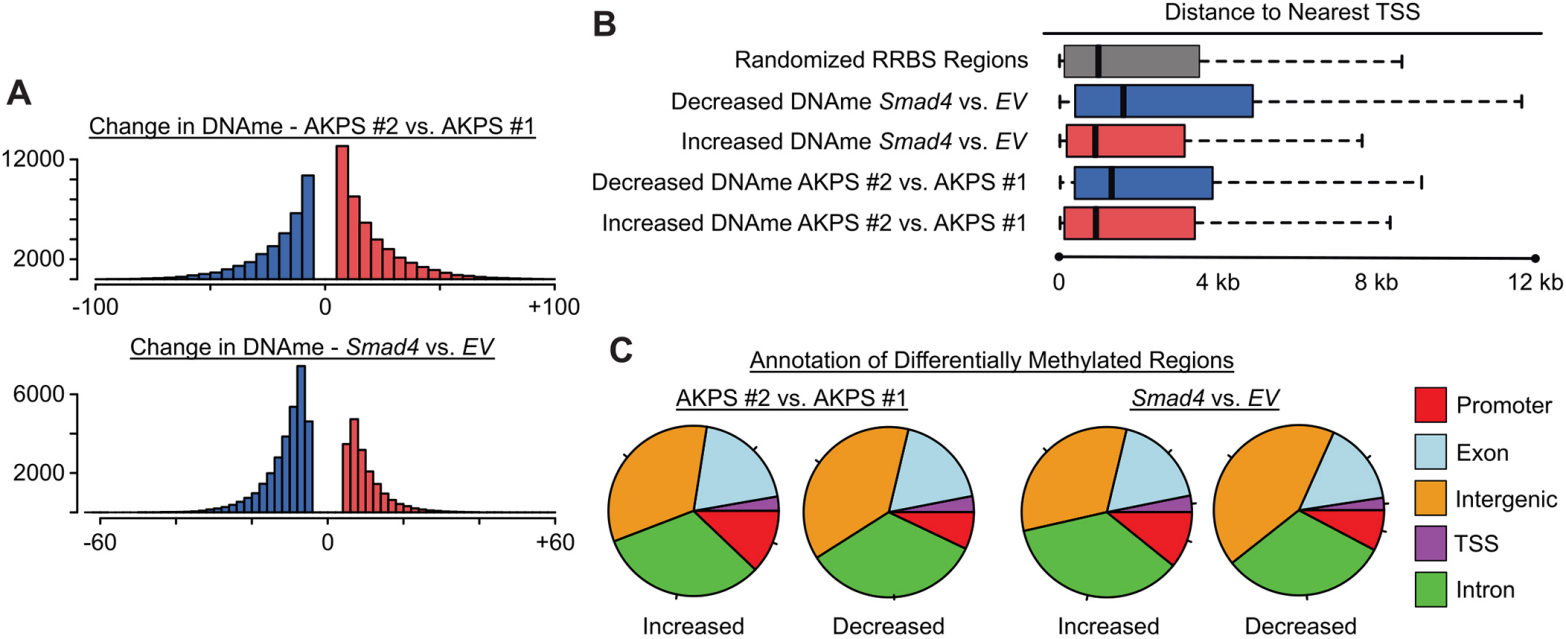
***Smad4* is a determinant of DNA methylation.** (A) Histograms indicating the frequency of differentially methylated regions binned according to changes in DNA methylation (DNAme; *x*-axis) in the two AKPS mouse model-derived CCA lines described in Fig.4. Increases (red) and decreases (blue) of DNAme were scored for AKPS *#*2 versus AKPS *#*1 (top) and for *Smad4* re-expression empty vector (*EV*) (bottom). (B) Boxplots indicate the distance (in kb) of differentially methylated regions relative to the nearest transcriptional start site (TSS). Regions from ‘Decreased DNAme *Smad4* vs. *EV*’ were found at a greater distance from the nearest TSS, suggesting a differential effect of *Smad4* on DNAme distal to promoter regions. (C) Differentially methylated regions were analyzed based on sample type (AKPS #2 vs AKPS #1; two pie charts on left) or *Smad4* re-expression [*Smad4* vs. *EV*; two pie charts on right) and further determined based on increased or decreased DNAme. Regions were then annotated and color-coded based on genetic context.

CCA is a heterogeneous disease at the genetic and epigenetic levels (Farshidfar et al., 2017; Goeppert et al., 2019; Jusakul et al., 2017). While *Smad4* has been described to impact methylation of several gene loci, its impact on epigenetic gene control in the setting of cancer is unknown. Reduced representation bisulfite sequencing (RRBS) on the same TGFβ-treated AKPS cell lines (with *EV* or *Smad4*) revealed that genome-wide DNA methylation (DNAme) levels were highly similar across samples, with significant differences between each AKPS cell line that are independent of the *Smad4* status (Fig. 5A; Fig. S6). *Smad4* re-expression led to both increased and decreased DNAme, with nearly double the number of affected regions showing a decrease than an increase in DNAme (14,478 vs 7853) (Fig. 5A). Consistently, 35% of regions with decreased DNAme were conserved between the two AKPS replicate cell lines, compared with 24% of regions in which DNAme was increased. *Smad4*-dependent regions with decreased DNAme were found at a greater distance from the nearest transcriptional start site (TSS) than other regions, suggesting a differential effect of *Smad4* on DNAme distal to promoter regions (Fig. 5B). By comparing separate AKPS cell lines and distinguishing between intact and deficient *Smad4*, sites of increased and decreased DNAme were mapped across genomic locations, and defined as TSS, promoter, exon, intron or intergenic. We found similar distributions of changes across all elements among our four comparisons with the exception of *Smad4*-decreased DNAme, which was enriched in intergenic regions (Fig. 5C). These studies demonstrate that *Smad4* impacts on DNAme, with decreases in intergenic DNAme as the most impacted in our CCA models.

## DISCUSSION

Chronic tissue injury is a key epidemiologic risk factor for hepatobiliary cancers. In this present study, we tested the impact of the most common mutations in human CCA on the ductular reaction – a key event in injury response and initiation of CCA development – and identified *Smad4* as a key suppressor of cholangiocyte proliferation. We further demonstrated that deletion of *Smad4* in murine hepatobiliary cancer models exacerbates related pathologies of CCA and biliary hamartomas, indicating that *Smad4* has a complex function in suppressing these processes. Expression analyses of reactive cholangiocytes and mouse-derived CCA lines suggest that *Smad4* may act via several established and novel processes including the cell cycle, the proto-oncogene protein MYC and oxidative phosphorylation, to regulate the proliferative injury response and CCA development. Activation of this pathway has been shown in human disease settings, where the TGFβ1 ligand itself is upregulated among preneoplastic lesions and CCA, while the receptor is present on normal biliary epithelium and retained through cancer development (Zen et al., 2005). Correspondingly, cyclin D1 expression increased as the tissues became more neoplastic. Zen et al. further confirmed the importance of the pathways by using human cultured cholangiocytes and demonstrating that these cells and cyclin D1 levels are responsive and suppressed by TGFβ treatment.

The ductular reaction is a key feature of states of biliary injury, such as infection by liver flukes; thus, it is possible that SMAD4 is dispensable in the uninjured state but becomes an essential tumor suppressor in controlling the proliferative events associated with ductular reactions induced by injury (Lee et al., 1997). The established protective role of the upstream TGFβ receptor in suppressing biliary carcinogenesis is consistent with this (Mu et al., 2016). In our present study, we established SMAD4 as a key suppressor of the proliferative response following biliary injury and as a tumor suppressor in hepatobiliary cancer models. We further linked these functions by demonstrating significant overlap in the pathways governed by *Smad4* in both reactive cholangiocytes and mouse CCA lines. Interestingly, mutations in *SMAD4* are strongly associated with liver-fluke-related intrahepatic CCA in humans, suggesting that, in this specific pathogenic context, inactivating *SMAD4* is especially advantageous to tumor development. This observation may also be related to the cellular origins of the CCA. As we and others have previously demonstrated, intrahepatic CCA in mice can be cholangiocyte-derived or hepatocyte-derived (Fan et al., 2012; Hill et al., 2018b; Sekiya and Suzuki, 2012). The predominance of *SMAD4* mutations in both liver-fluke-related intrahepatic CCA and extrahepatic CCA suggests the biliary tree as the originating cellular compartment in these contexts. Thus, the newly identified role for *Smad4* in suppressing cholangiocyte proliferation in mice suggests *SMAD4*-mutant CCA is more strongly associated with cholangiocyte-derived CCA in humans. We have previously shown that TP53 mutations in hepatocytes create lineage plasticity. It is possible that SMAD4 loss may also enable this. While at this point, we can only hypothesize, we anticipate that future studies beyond the scope of this article, will help clarify this observation. In addition to the underlying tumor genetics, understanding the cellular origins of these tumors may prove to have valuable therapeutic and prognostic significance.

A number of signaling pathways have previously been established as key regulators of the ductular reaction, such as Notch, Wnt/β–catenin and Hippo/Yap, as well as less well-characterized roles for Hedgehog, NF-κB, IGF signaling and integrin signaling (Jors et al., 2015; Kim et al., 2015; Okabe et al., 2016; Omenetti et al., 2008; Sokolovic et al., 2013; Yanger et al., 2013; Yimlamai et al., 2014). Furthermore, interactions among these various pathways have been identified, suggesting that they are likely to form a complex regulatory network. Here, we establish the classic tumor suppressive TGFβ/SMAD4-signaling axis as a novel regulator of the ductular reaction. Our analyses revealed known and potentially novel downstream processes that might mediate its function, including cell cycle, MYC signaling and oxidative phosphorylation. Our study provides links between this molecular pathway, ductular proliferation, cancer development and cell-cycle controls in a cholangiocyte/cholangiocarcinoma system. Among the novelties are the creation of a new CCA model system with compound KRAS/TP53/SMAD4 mutant mice and the characterization of the derivative cell lines. Last, we provide novel data that *Smad4* is a determinant of genome-scale DNAme patterns in CCA. This effect might be a consequence of direct interaction of *Smad4* with methylation complexes on DNA; it is also possible, however, that these changes are reflective of proliferative/cell-cycle changes in general, which are provoked by *Smad4* loss. Methylation state has recently been recognized as a distinguishing feature of both etiologically and molecularly distinct subtypes of CCA (Farshidfar et al., 2017; Goeppert et al., 2019; Jusakul et al., 2017). For example, liver-fluke-related CCAs are strongly associated with hypermethylation of promoter CpG islands. The TGFβ/SMAD4-signaling axis has previously been linked to epigenetic regulation of specific genomic loci including *RUNX1T1* and *VAV1* (Cardenas et al., 2014; Huang et al., 2017; Yeh et al., 2011). Here, we demonstrate that *Smad4* inactivation in CCA may have broad impact on genomic methylation patterns; the specific mechanism(s) by which this occurs as well as the biological and therapeutic consequences will, ultimately, be important to understand more fully.

## MATERIALS AND METHODS

### Mice

All animal studies were conducted in accordance with the AAALAC accredited University Committee on Animal Resources (UCAR). Mice were of mixed genetic background. All mouse strains (*Alb-Cre*, *Arid1a^fl/fl^*, *Kras^LSL-G12D^*, *Smad4^fl/fl^*, and *Tp53^fl/fl^*) used have been previously characterized (Bardeesy et al., 2006; Gao et al., 2008; Jackson et al., 2001; Jonkers et al., 2001; Postic et al., 1999). In survival studies, mice were monitored for signs of illness, including abdominal bloating, diminished activity and/or poor grooming. For liver injury studies, mice were fed a 0.1% of chow 3,5-diethoxycarbonyl-1,4-dihydrocollidine (DDC) diet ad libido for 2 weeks as depicted in the diagram from Fig. 1A and Fig. 2A (Custom Animal Diets, AD5001). For the BDL injury model, the protocol outlined by Tag et al. was closely followed (Tag et al., 2015). Briefly, mice were anesthetized using isoflurane and the common bile duct was surgically ligated. Buprenorphine was provided for the first 3 days following surgery. In all experiments, littermates were used as controls where possible.

### Histology

Tissue was fixed in 10% formalin, paraffin embedded, cut into 4-μm sections and put on slides. For immunohistochemistry (IHC) and immunofluorescence (IF), sections were deparaffinized, underwent antigen retrieval in a citrate buffer for 30 min and blocked for 4 min in Background Sniper (Biocare Medical, cat. no. BS966). Sections were incubated with primary antibodies overnight at 4°C and secondary antibodies for 2 h at room temperature. A Mouse on Mouse kit (Vector Laboratories, cat. no. BMK-2202) was used if appropriate. IHC sections were then either incubated by using the appropriate ABC kit followed by VECTOR Red or DAB substrate kits (Vector Laboratories; cat. no. AK-5000 for Vectastain ABC AP Kit, cat. no. PK-4000 for Vectastain ABC HRP Kit, cat. no. SK-5100 for VECTOR Red Alkaline Phosphatase Substrate Kit, cat. no. SK-4100 for DAB Peroxidase (HRP) Substrate Kit)), and then stained with hematoxylin, rehydrated and mounted with Cytoseal kits (VWR, cat. no. 8310-4). IF sections were incubated with Hoechst 33342 (Invitrogen, cat. no. H3570) for 45 min at room temperature, then mounted in ProLong Gold Antifade Mountant (Life Technologies, cat. no. P36930). IF was visualized using confocal microscopy. All histology was reviewed by gastrointestinal pathologist Dr Christa Whitney-Miller. All tumors were stained with CK7 and ARG1 to determine tumor differentiation. Quantification of histology data and IHC was performed using software on the Aperio Versa system or ImageJ (https://imagej.net/ij/). For collagen quantification, mouse liver sections were stained with Picosirius Red according to manufacturer instructions (Polyscientific R&D). The following primary antibodies were utilized: rabbit anti-cleaved caspase 3 (Cell Signaling #9664, dilution 1:50), rabbit anti-Ki67 (Abcam #ab66155, dilution 1:50), rat anti-KRT19 (DSHB TROMAIII, dilution 1:50), rabbit anti-PanCK (Dako #Z0622, dilution 1:500), rabbit anti-phospho-SMAD2 (Abcam #ab219598, dilution 1:100), rabbit anti-Arginase I (Thermo Fisher Scientific #PA5-29645, dilution 1:500), and rabbit anti-KRT7 (Abcam #ab181598, dilution 1:500). Secondary antibodies include biotinylated goat anti-rabbit (Vector #BA-1000), Alexa-Fluor-488 goat anti-rabbit (Thermo Fisher Scientific #A-11008), Alexa-Fluor-555 goat anti-rat (Thermo Fisher Scientific #21434).

### qPCR

RNA was extracted from frozen liver tissue or cell lines using the RNeasy Mini Kit (Qiagen). cDNA was created using a cDNA reverse transcriptase kit (Applied Biosystems, cat. no. 4368814). All qPCR reactions were performed in triplicates, using SYBR green master mix (Bio-Rad). Expression levels were normalized to *Rhoa*. Specific primer sequences can be found in Table S3.

### Western blotting

Culture plates were washed with PBS; cold RIPA buffer (Cell Signaling, cat. no. 9806) containing protease inhibitor (Sigma, cat. no. P8340) and phosphatase inhibitors (Sigma, cat. nos P5726 and P0044) was added directly to the plate. Plates were scraped on ice, the lysate was spun down (16,000 ***g*** for 15 min in a 4°C pre-cooled centrifuge), and the supernatant was collected. Protein concentrations were determined in a Bradford assay. 5× loading buffer [5× solution of 250 mM Tris·HCl pH 6.8, 10% SDS/30% glycerol (v/v), 10 mM DTT, 0.05% (w/v) Bromophenol Blue (w/v)] was added to the sample and heated at 95°C for 10 min. The proteins were separated using gel electrophoresis and transferred to a PVDF membrane at 300 mA for 3 h at 4°C. The membrane was incubated in 5% milk in TBS 0.1% Tween (TBST) blocking solution for 30 min at room temperature. Primary antibodies were diluted in 5% milk TBST and incubated with the membrane overnight at 4°C. Following three washes with TBST, the membrane was incubated with HRP-conjugated secondary antibody in 5% milk TBST for 2 h at room temperature. Following three washes, the membrane was incubated with ECL Western Blotting Reagent and developed.

### Liver digestion and FACS for reactive cholangiocyte isolation

While a mouse was anesthetized, its liver was perfused via the portal vein with perfusion buffer [1 mM EGTA in Hanks’ balanced salt solution (HBSS, Gibco, cat. no. 14175-095)]. After removing the liver, it was minced in a dish with digestion buffer [40 μg/ml Liberase TM (Sigma, cat. no. 05401119001), 5 mM CaCl_2_ in HBSS] and then shaken in digestion buffer at 37°C for 30 min. This mixture was filtered through a 70 μm strainer, and the remaining undigested tissue underwent additional digestion (20-30 ml digestion buffer in total) for 30 min, after which it was again filtered through the 70 μm strainer. This then underwent three spins (50 ***g*** for 5 min) to pellet hepatocytes, and the supernatant was pelleted at 300 ***g*** for 5 min to isolate the nonparenchymal cells containing reactive cholangiocytes. Nonparenchymal cells were incubated in blocking buffer (3% FBS in PBS) for 20 min on ice, followed by incubation with antibodies against EPCAM (1:100, Biolegend #118206), CD45 (1:100, Biolegend #103116), and TER119 (1:100, eBioscience #11-5921-82). After three washes with blocking buffer, cells were incubated with TO-PRO-3 viability dye (Thermo Fisher Scientific, cat. no. T3605) at a 1:1000 dilution. Fluorescence-activated cell sorting (FACS) was performed using as FACSAria II for the EPCAM+/CD45-/TER119-/TO-PRO-3- population, and cells were sorted into RLT Plus lysis buffer (Qiagen, cat. no. 1053393).

### Preparation, sequencing and analysis of RNA from Smad4-perturbed reactive cholangiocytes

Ductular reactive cell (DRC) RNA preparation, sequencing, and preliminary analyses were performed by the University of Rochester Genomics Research Center. RNA was isolated using a RNeasy Plus Micro Kit (Qiagen, cat. no. 74034). For qPCR analyses, the Nugen Ovation PicoSL WTA System (Tecan Genomics, cat. no. 3312) was used to generate amplified cDNA. For RNA-sequencing, a Nextera cDNA library was prepared using the SMART-Seq v4 Ultra Low Input RNA Kit (Takara Bio, cat. no. 634888). Sequencing was performed on an Illumina HiSeq 2500 system (https://support.illumina.com/sequencing/sequencing_instruments/hiseq_2500.html) using single-end 100 bp read lengths. Sequence reads were cleaned and adapter trimmed using trimmomatic-0.36 (Bolger et al., 2014, https://github.com/usadellab/Trimmomatic) before mapping each sample individually to the mouse reference genome (GRCm38.p5, primary assembly+GENCODE M12 annotation) with STAR2.5.2b (Dobin et al., 2013, https://github.com/alexdobin/STAR). Raw read counts were obtained using featurecounts from the subread1.5.0p3 package (Liao et al., 2013, https://subread.sourceforge.net) and GENCODE M12 mouse gene annotations using only uniquely aligned reads (default) and including multi-mapping reads (-M). DESeq2-1.14.1 within R-3.3.2 (Love et al., 2014, https://doi.org/10.18129/B9.bioc.DESeq2) was used to perform data normalization and differential expression analysis with an adjusted *P*-value threshold of 0.05 on each set of raw expression measures. Data were visualized using the GENE-E platform (https://software.broadinstitute.org/GENE-E). Pathway analyses were performed using the pre-ranked tool in GSEA software (https://www.gsea-msigdb.org/gsea).

### Cell line RNA preparation for sequencing and analysis

RNA was extracted from cell lines using the RNeasy Mini Kit (Qiagen). cDNA was created using a cDNA reverse transcriptase kit. Data was visualized using the GENE-E platform. Pathway analyses were performed using the pre-ranked tool in GSEA software.

### Liver digestion and flow cytometric analysis of immune infiltrate

Livers were removed from mice, minced, and digested with collagenase D for 45 min. Single cell suspensions were blocked in Fc Block and incubated with appropriate antibodies for 30 min. Flow analysis was performed on 12-color LSRII with FlowJo software (https://www.flowjo.com). The following antibodies were used anti-CD45 (APC/Cy7; Biolegend, clone 30-F11, #103115), anti-CD11b (APC; Biolegend, M1/70, #101211), anti-Ly6C (PE/Cy7; Biolegend HK1.4, #128017), anti-Ly6G (Pacific Blue; Biolegend, clone 1A8, #127611).

### Cell culture

All cell lines were maintained in DMEM with 10% FBS. For cell lines containing integrated gene expression constructs with puromycin resistance, puromycin was removed at least 24 h prior to experiments. To derive primary cell lines, primary tumors were minced and digested in collagenase (1 mg/ml) in DMEM (no FBS) for 30 min and plated in DMEM with 10% FBS and antibiotics kanamycin (100 µg/ml), streptomycin (100 µg/ml), penicillin (100 units/ml) and gentamicin (2 µg/ml). Cells were maintained in medium supplemented with antibiotics for at least three passages. Experiments in which cells were treated with TGFβ1, the latter was provided at 20 ng/ml in DMEM with 1% FBS. Cells were changed to low FBS medium 16 h prior to treatment. RNA was harvested 48 h after treatment.

### Lentivirus expression constructs

shRNA targeting mouse *Smad4* was obtained from the RNAi Consortium/Dharmacon. sh*Smad4* #1 corresponds to clone TRCN0000025881 (catalog #RMM3981-201756188), and sh*Smad4* #2 corresponds to clone TRCN0000025885 (catalog #RMM3981-201756192). The lentiviral construct pLenti-C-Myc-DDK-P2A-Puro expressing murine *Smad4* was generated by Origene (catalog #MR208755L3). To generate the empty vector, the pLenti-C-Myc-DDK-P2A-Puro-*Smad4* plasmid was digested with EcoRI and XhoI to excise the *Smad4*-encoding region. The linearized vector was purified after gel electrophoresis separation. An insert was designed with overhangs compatible to EcoRI and XhoI. The insert and linearized vector were ligated with T4 DNA Ligase kit, and the ligation mixture was transformed into One Shot Stbl3-competent *E. coli* cells. The plasmid was generated from resulting colonies and verified through Sanger sequencing for the insert sequence.

### Lentivirus generation and target cell infection

3 μg of packaging plasmid Pax2 and 1.5 μg of envelope plasmid VSV-G were mixed with 2 μg of the appropriate transfer plasmid in 500 μl DMEM (no FBS). This plasmid mixture was then combined with a mixture of 40 μl Lipofectamine 2000 (Invitrogen, cat. no. 11668-019) in 500 μl DMEM (no FBS) and incubated at room temperature for 15 min. The plasmid/Lipofectamine mixture was added to HEK293 T cells at ∼50% confluency in a 10-cm dish. 16 h later, fresh DMEM supplemented with 10% FBS was added to HEK293 T cells. 24 h after that, the medium containing lentivirus was collected and filtered through a 0.45 μm filter. Target cells were ∼50% confluent at time of infection. To infect, virus containing medium was added to target cells. Polybrene (8 μg/ml, Tocris Bioscience, cat. no. 77-111-0) was added dropwise to the virus medium/target cell plate. This was done in three rounds, each lasting 2 h. After at least 24 h of recovery, cells containing the expression construct were selected for in the appropriate concentration of puromycin-containing medium over 5 days, at which time all mock-treated cells were dead.

### DNA methylation sample preparation and analyses

Library construction and DNA methylation (DNAme) bioinformatics analysis. Purified DNA samples were digested with restriction enzyme MspI. Following digestion, Klenow Fragment was utilized to create 3′A overhangs. DNAs were subsequently column purified, and then ligated to methylated Illumina PE Adapters. Ampure SPRI beads were used to purify products prior to sodium bisulfite conversion and amplification to generate libraries. Sequencing was performed on an Illumina NextSeq550 system using a single-end 100 bp protocol (https://www.illumina.com/systems/sequencing-platforms/nextseq.html). RRBS sequencing data were aligned to the mouse mg38 genome using the Bismark pipeline with special attention to RRBS specific issues, as noted in the Bismark User Guide and the Bismark RRBS Guide (Krueger and Andrews, 2011, https://www.bioinformatics.babraham.ac.uk/projects/bismark). CpGs were then further parsed and analyzed using Bismark output tables in R. Briefly, only CpGs with read coverage >8 across all samples were considered scoreable for downstream analysis. CpGs were then grouped into regions when they occurred within 50 bp of one another. Regions with more than 5% absolute change in methylation relative to control samples were classified as differentially methylated. RRBS regions were annotated using Homer AnnotatePeaks function (http://homer.ucsd.edu/homer/ngs/annotation.html), and the output of Homer was then used to assess CpG density, and proximity to nearest transcription start site. All figures describing RRBS data were generated using standard graphing and plotting functions in R.

### Statistics

Statistical analyses, except for sequencing data, were performed using Prism GraphPad Software. Survival was determined using the Kaplan-Meier method, and comparisons between genotypes were determined using the log-rank test. Animals that were found to have histological evidence of cancer were included as events, while those that died for reasons other than cancer were censored. Column graphs represent mean±standard deviation. Unless otherwise noted, the unpaired *t*-test with Welch's correction was used to determine statistical significance. Mann–Whitney tests were used for analysis of immune infiltrate in Fig. S2. The Fisher's exact test was applied in Fig. S3D,F. For RNA-sequencing data all references to the *P*-value refer to the possession-adjusted (PAdj) value. The following abbreviations/symbols were used throughout this document to indicate relative significance: *P<0.05, **P<0.01, ***P<0.0001.

## Supplementary Material

10.1242/dmm.050358_sup1 Supplementary information
