# Supplementary material for: Smad4 restricts injury-provoked biliary proliferation and carcinogenesis
Source: Dis Model Mech. 2024 Feb 28;17(6):dmm050358. doi: 10.1242/dmm.050358 (PMC10924230; doi:10.1242/dmm.050358)
Supplement: Supplementary information [file dmm-17-050358-s1.pdf]

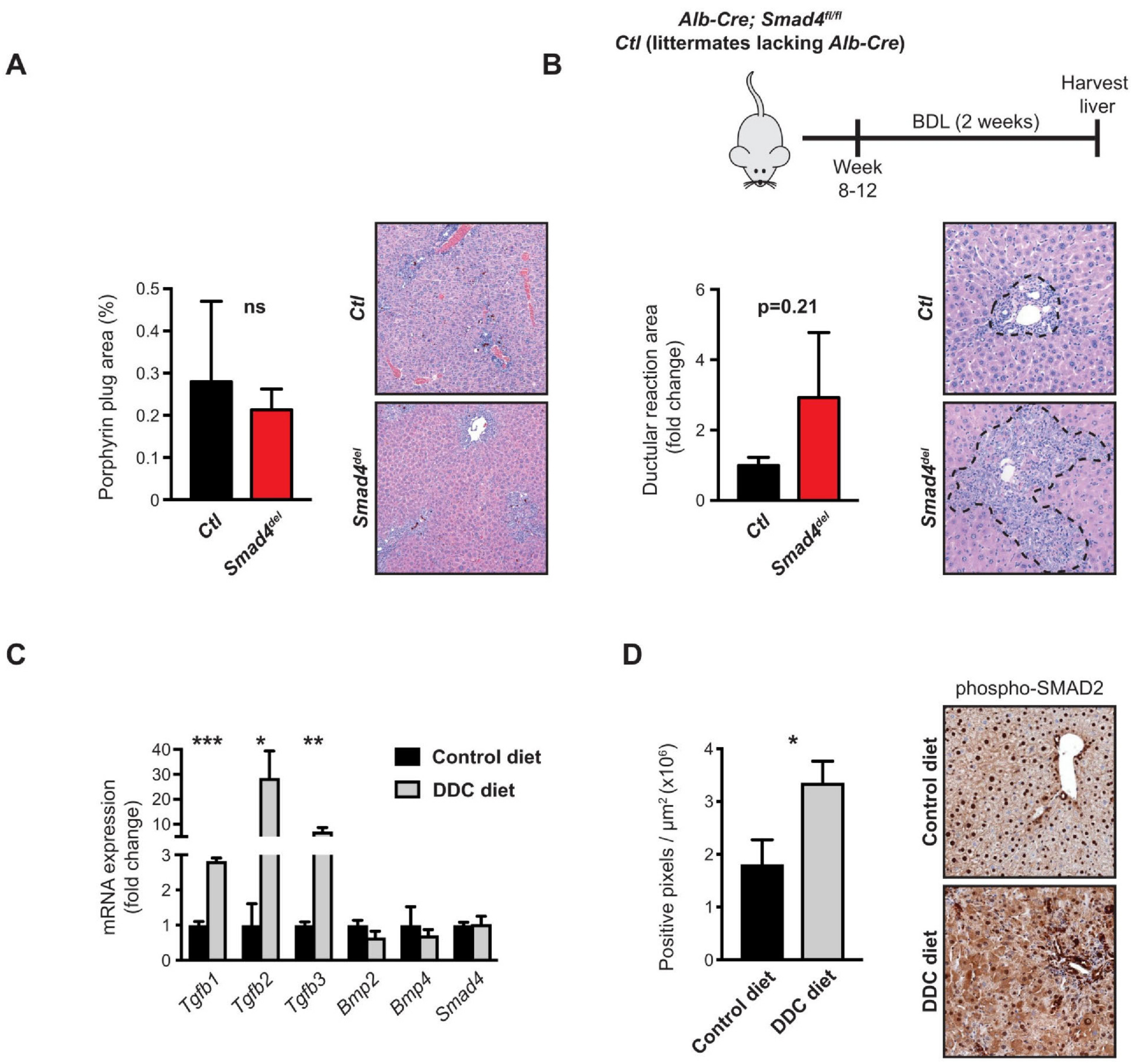
A
B
Alb-Cre; Smad4fl/fl
Ctl (littermates lacking Alb-Cre)
Harvest liver
BDL (2 weeks)
Week 8-12
Porphyrin plug area (%)
ns
Ctl
Smad4del
Ductular reaction area (fold change)
p=0.21
C
D
mRNA expression (fold change)
***
*
**
Control diet
DDC diet
Tgfb1
Tgfb2
Tgfb3
Bmp2
Bmp4
Smad4
Positive pixels / µm2 (x10-6)
phospho-SMAD2
Control diet
DDC diet

Fig. S1. (A) *Smad4^{del}* livers do not accumulate more porphyrin plugs after two weeks of DOC. Quantification of porphyrin plug positive area compared to total liver area. Representative images (magnified 100x) are shown on the right. Random fields (at least 3 fields/mouse) were used for analyses. (B) *Smad4* suppresses ductular reaction expansion in the bile-duct-ligation (BDL) model. Surgical bile-duct ligations were performed on *Smad4^{del} and Ctl* mice and livers were harvested after two weeks of injury, n=3-4 mice per genotype. Quantification of BDL ductular reaction area compared to total liver area is shown. Random 40x fields (at least 8 fields/mouse) were used for analyses . Representative images of ductular reactions (surrounded by dashed lines, magnified 200x) are shown on the right. There is a wider range of ductular expansion in the *Smad4^{del} cohort* likely due the variable effect of ligation. (C) mRNA expression levels assessed by qPCR of TGF13 family-related genes in DOC diet liver compared to normal liver tissue showing increased expression of TGFβ ligands. Expression was normalized to *Rhoa,* n=3 mice per cohort. (D) Quantification showing increased nuclear phospho-SMAD2 staining in DOC diet liver compared to control. Representative images (magnified 200x) are shown on the right. ns, not significant, *p<0.05, **p<0 .01, ***p<0.001, Bar=50 μm in A, Bar=25 μm in B and D.

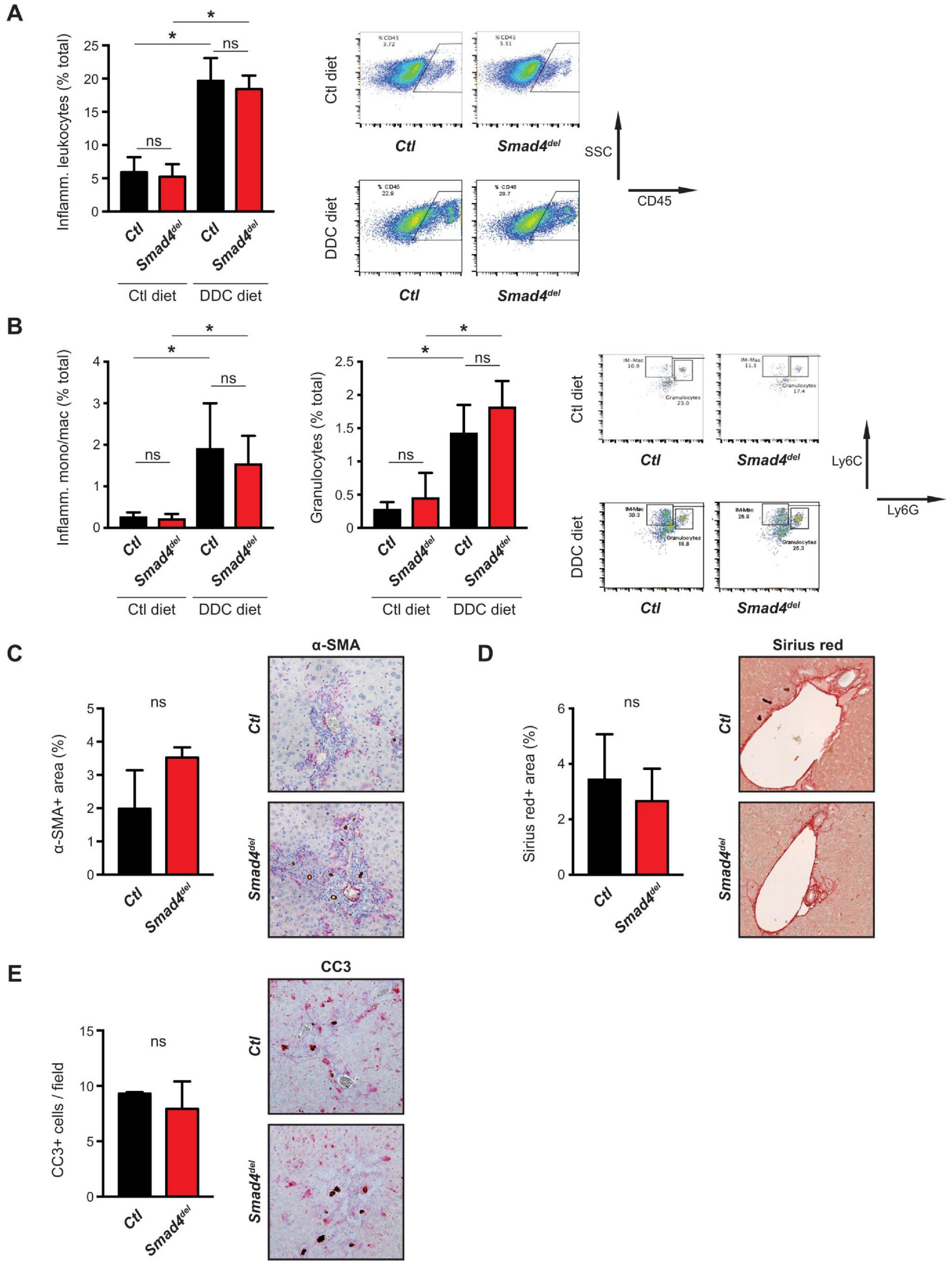

Disease Models & Mechanisms • Supplementary information

Fig. S2. A) Quantification of inflammatory leukocytes in control and injured (DOC diet) livers of *Ctl* and *Smad4*^{del} mice showing no significant difference . Livers from mice on control diet (n=3 mice per cohort) and DOC diet (n=6 mice per cohort) were digested and analyzed for cells staining positive for the immune marker CD45. Representative FAGS plots are shown on the right. (B) Quantification of inflammatory monocytes/macrophages and granulocytes in control and injured (DOC diet) livers of *Ctl* and *Smad4*^{del} mice showing no significant difference. Livers from mice on control diet (n=3 mice per cohort) and DOC diet (n=6 mice per cohort) were digested and analyzed for cells staining positive for the following immune markers CD45+/CD11b+/Ly6C-high/Ly6G-low for monocytes/macrophages and CD45+/CD11 b+/Ly6C-high/Ly6G-high for granulocytes. Representative Ly6C/Ly6G plots are shown on the right. (C) Quantification of area staining positive for the myofibroblast marker aSMA compared to total liver revealing no significant difference . Random 40x fields were used for quantification , n=3 mice per cohort with 3 fields per mouse. Representative images are shown on the right (magnified 200x). (D) Quantification of collagen area staining positive for Sirius red showing no significant difference, n=3 mice per cohort . Representative images are shown on the right (magnified 100x). (E) Quantification of cells staining positive for apoptosis cleaved-caspase 3 (CC3) revealing no significant difference .Random 200x images were used for quantification, n=2-3 mice per cohort with 5 fields per mouse. Representative images are shown on the right (magnified 200x). ns, not significant ,*p<0 .05, Bar=25 µm in C and E, Bar=50 µm in D.

Disease Models & Mechanisms • Supplementary information

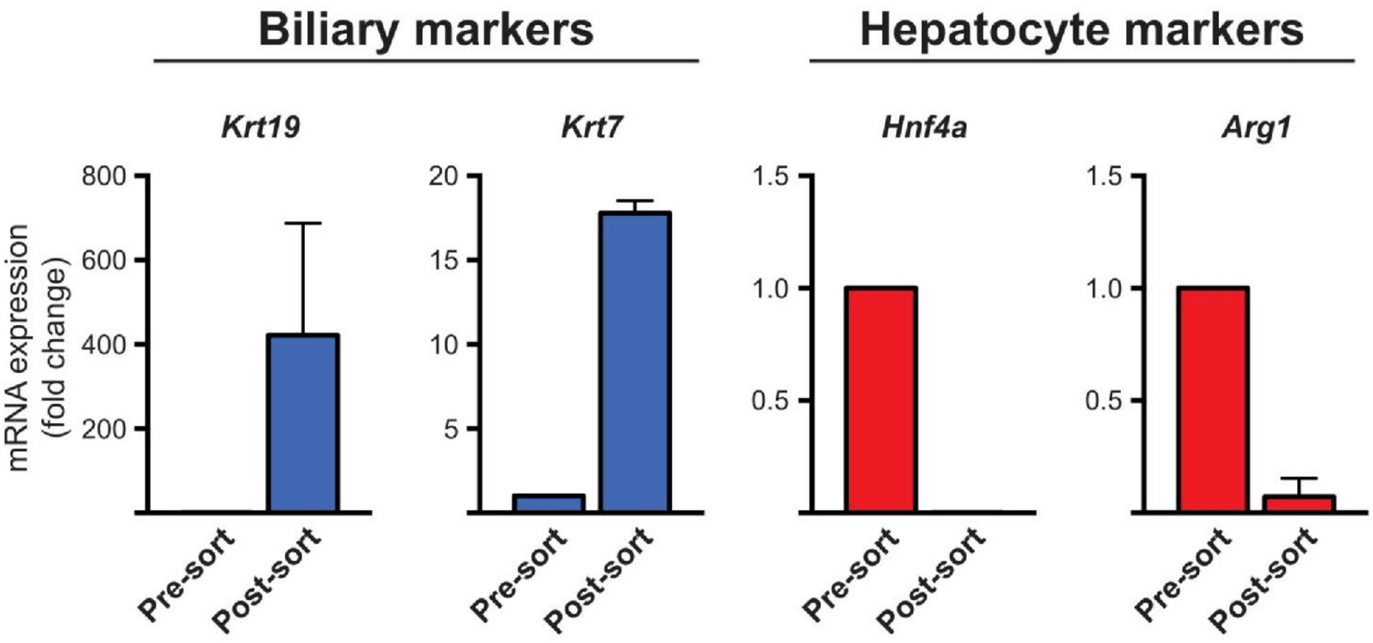

Fig. S3. FACS-isolation enriches for reactive cholangiocytes. mRNA expression levels assessed by qPCR of hepatocyte and biliary markers in samples taken before the sort and after the sort, demonstrating enrichment of the biliary compartment.

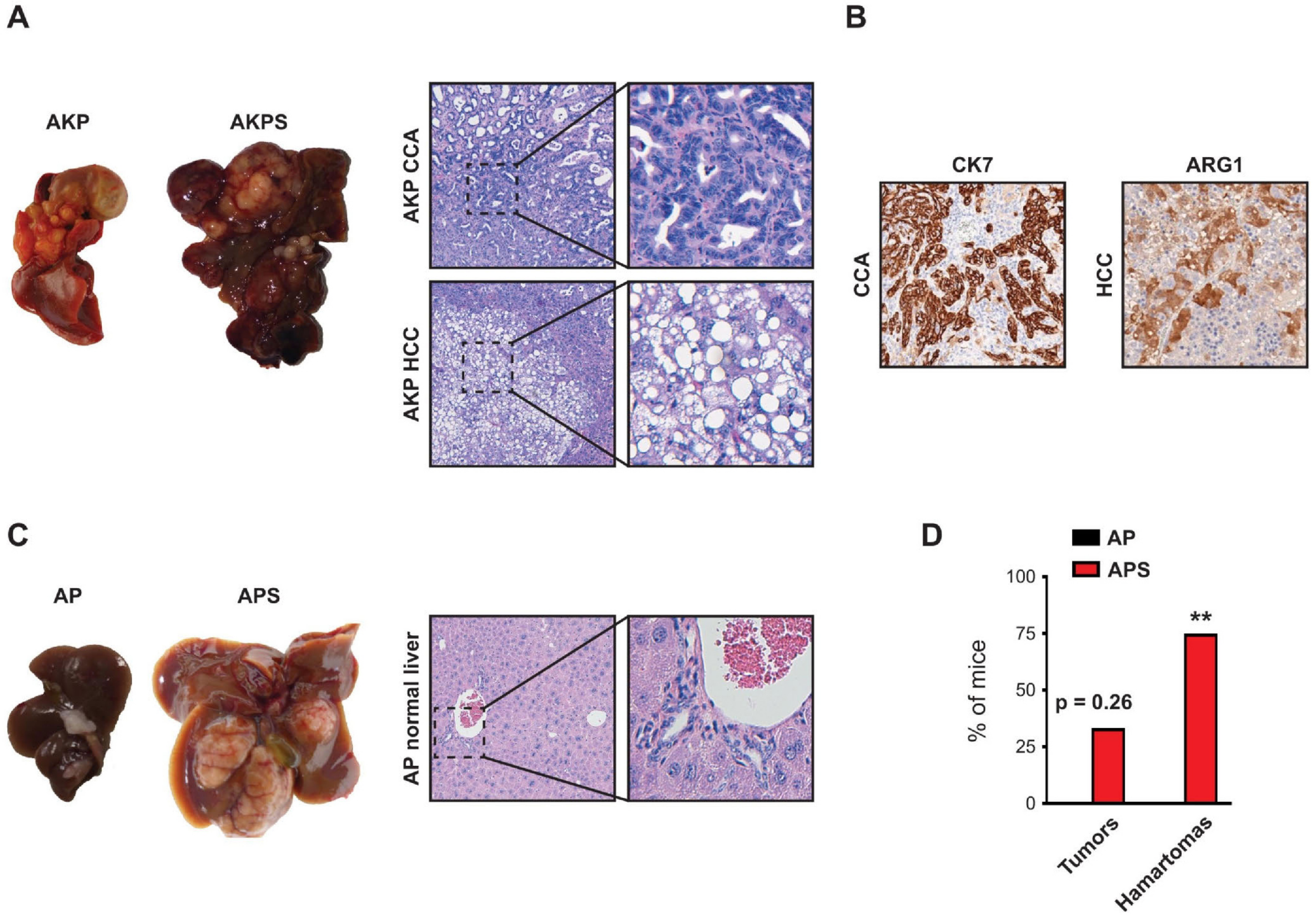

Fig. S4. (A) Macroscopic images of AKP and AKPS livers with representative histology images of control AKP CCA and HCC (magnified 100x with selected boxed areas shown enlarged on the right). (B) Representative images of positive CK7 staining and ARG1 staining in murine CCA and HCC, respectively (magnified 100x). (C) Macroscopic images of AP and APS with representative histology images of control AP livers with normal histology (magnified 100x with selected boxed area shown enlarged on the right). (D) Quantification of tumors and hamartomas in AP and APS mice. *p<0.01, Bar=50 µm.

Disease Models & Mechanisms • Supplementary information

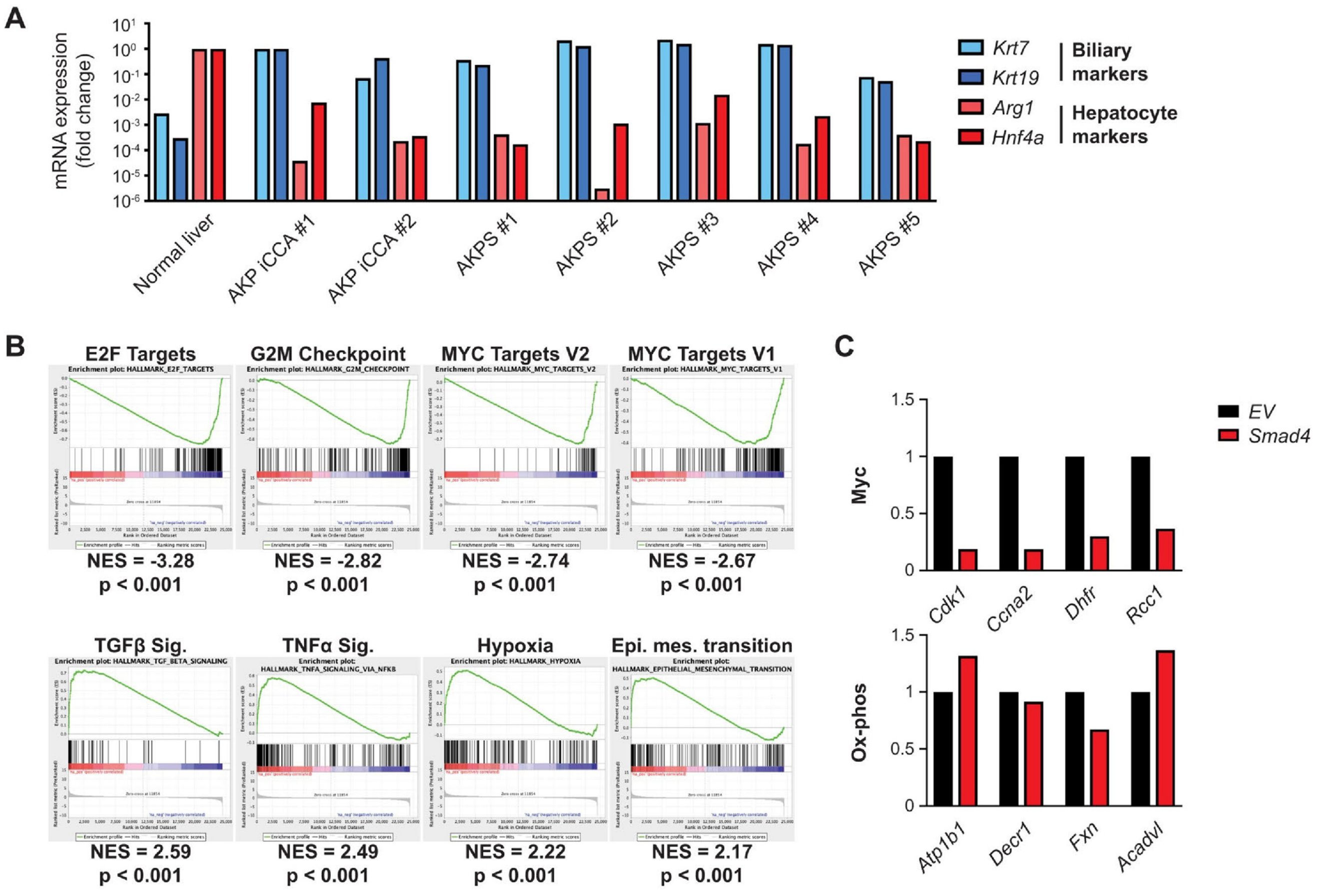

Fig. S5. (A) mRNA expression levels assessed by qPCR to characterize the differentiation state of AKPS cell lines *in vitro.* Included are normal liver to provide a representative hepatocellular signature and AKP cell lines derived from CCA (AKP iCCA #1 and #2) to represent a biliary signature. AKPS cell lines #1-5 show expression of biliary markers. Expression was normalized to *Rhoa.* (B) Gene set enrichment analysis (GSEA) of genes differentially expressed in *Smad4*-restored CCA cell lines. Enrichment plots from the Hallmarks Gene Set Collection are shown for the top five gene sets most negatively correlated (top row) in genes downregulated in *Smad4*-restored CCA cell lines or most positively correlated (bottom row) in genes upregulated in *Smad4*-restored CCA cell lines. (C) mRNA expression levels assessed by qPCR of MYC target genes and Ox-Phos related genes derived from the leading edge analysis of reactive cholangiocyte GSEA. Expression was normalized to *Rhoa.* Ox-Phos, oxidative phosphorylation; EMT, epithelial mesenchymal transition; NES, normalized enrichment score.

Disease Models & Mechanisms • Supplementary information

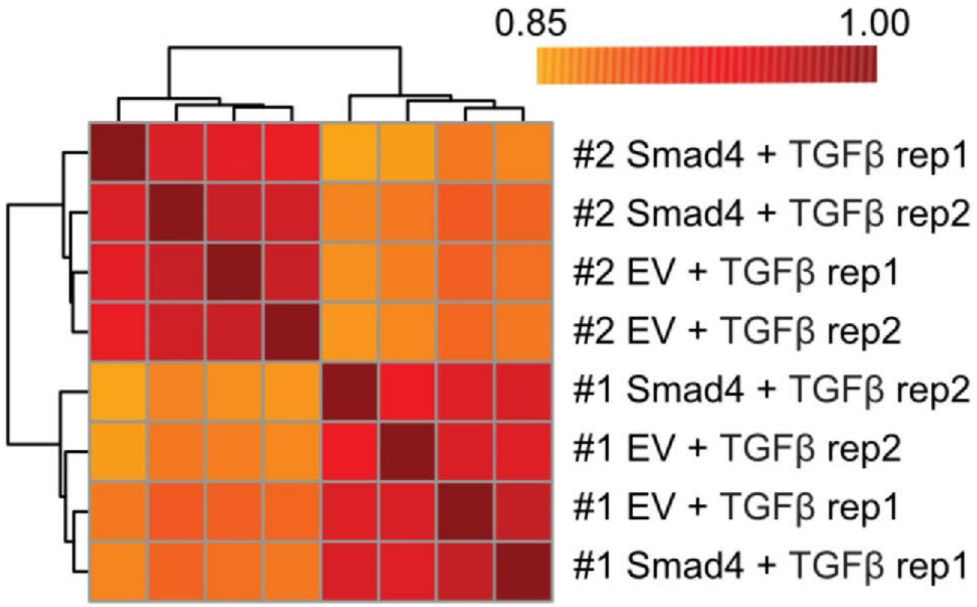

Fig. S6. Hierarchical clustering of DNAme changes in *Smad4* restored CCA cell lines. Pearson correlation coefficients were determined for pairwise comparisons of all genome-wide DNAme (RRBS) datasets. Hierarchical clustering was then applied to group samples to distinguish similarity in datasets. A colored heatmap scale is shown to indicate Pearson R values.

Table S1. Summary of AKPS and AKP liver tumors

Mouse Number	Genotype (*Alb-Cre*)	Age at death* (weeks)	Liver Histology
2797	$Kras^{LSL\text{-}G12D}$ $Tp53^{fl/+}$ $Smad4^{fl/fl}$	24	CCA, HCC, BilIN
A3333	$Kras^{LSL\text{-}G12D}$ $Tp53^{fl/+}$ $Smad4^{fl/fl}$	45	CCA, HCC
A3368	$Kras^{LSL\text{-}G12D}$ $Tp53^{fl/+}$ $Smad4^{fl/fl}$	51	CCA, HCC, BilIN, BH
A3370**	$Kras^{LSL\text{-}G12D}$ $Tp53^{fl/+}$ $Smad4^{fl/fl}$	60	-
A3474	$Kras^{LSL\text{-}G12D}$ $Tp53^{fl/+}$ $Smad4^{fl/fl}$	29	CCA, HCC, BH
A3722	$Kras^{LSL\text{-}G12D}$ $Tp53^{fl/+}$ $Smad4^{fl/fl}$	31	CCA, HCC, BH
A3723	$Kras^{LSL\text{-}G12D}$ $Tp53^{fl/+}$ $Smad4^{fl/fl}$	29	CCA, HCC, BilIN, BH
A3980	$Kras^{LSL\text{-}G12D}$ $Tp53^{fl/+}$ $Smad4^{fl/fl}$	47	CCA, HCC
A3981	$Kras^{LSL\text{-}G12D}$ $Tp53^{fl/+}$ $Smad4^{fl/fl}$	50	CCA, HCC, BilIN
A3984	$Kras^{LSL\text{-}G12D}$ $Tp53^{fl/+}$ $Smad4^{fl/fl}$	48	CCA, HCC, IPBN
2522	$Kras^{LSL\text{-}G12D}$ $Tp53^{fl/+}$	56	CCA, HCC
A2331	$Kras^{LSL\text{-}G12D}$ $Tp53^{fl/+}$	82	Normal
A2570	$Kras^{LSL\text{-}G12D}$ $Tp53^{fl/+}$	38	CCA, HCC
A2571	$Kras^{LSL\text{-}G12D}$ $Tp53^{fl/+}$	55	CCA
A3131**	$Kras^{LSL\text{-}G12D}$ $Tp53^{fl/+}$	65	-

* Due to disease burden
** Poor tissue quality

Table S2. Summary of APS and AP liver tumors

Mouse Number	Genotype (*Alb-Cre*)*	Liver Histology
2959	$Tp53^{fl/fl}$ $Smad4^{fl/fl}$	BH
2967	$Tp53^{fl/fl}$ $Smad4^{fl/fl}$	BH
4472	$Tp53^{fl/fl}$ $Smad4^{fl/fl}$	BH, IPBN
A341	$Tp53^{fl/fl}$ $Smad4^{fl/fl}$	BH
A343	$Tp53^{fl/fl}$ $Smad4^{fl/fl}$	BH
A424	$Tp53^{fl/fl}$ $Smad4^{fl/fl}$	BH
A441	$Tp53^{fl/fl}$ $Smad4^{fl/fl}$	Normal
A577	$Tp53^{fl/fl}$ $Smad4^{fl/fl}$	Normal
A683	$Tp53^{fl/fl}$ $Smad4^{fl/fl}$	CCA, HCC, BH
A684	$Tp53^{fl/fl}$ $Smad4^{fl/fl}$	HCC, BH
A689	$Tp53^{fl/fl}$ $Smad4^{fl/fl}$	CCA, HCC, BH
A1422	$Tp53^{fl/fl}$ $Smad4^{fl/fl}$	BH
A1424	$Tp53^{fl/fl}$ $Smad4^{fl/fl}$	CCA, HCC, BH
A1659	$Tp53^{fl/fl}$ $Smad4^{fl/fl}$	Normal
A1661	$Tp53^{fl/fl}$ $Smad4^{fl/fl}$	Normal
2670	$Tp53^{fl/fl}$	Normal
2865	$Tp53^{fl/fl}$	Normal
2931	$Tp53^{fl/fl}$	Normal
2935	$Tp53^{fl/fl}$	Normal
2946	$Tp53^{fl/fl}$	Normal
2972	$Tp53^{fl/fl}$	Normal
4322	$Tp53^{fl/fl}$	Normal
4349	$Tp53^{fl/fl}$	Normal

* Euthanized at time point

Disease Models & Mechanisms • Supplementary information

Table S3. Primer Sequences

Target mRNA	Forward Primer (5' to 3')	Reverse Primer (5' to 3')
Acadvl	CTACTGTGCTTCAGGGACAAC	CAAAGGACTTCGATTCTGCCC
Atp1b1	GCTGCTAACCATCAGTGAACT	GGGGTCATTAGGACGGAAGGA
Arg1	TTGGGTGGATGCTCACACTG	GTACACGATGTCTTTGGCAGA
Bmp2	CACACAGGGACACACCAACC	CAAAGACCTGCTAATCCTCAC
Bmp4	CGTCATTCCGGATTACATGAGGGA	CCTGGGATGTTCTCCAGATGTTCT
Ccna2	GCCTTCACCATTCATGTGGAT	TTGCTCCGGGTAAAGAGACAG
Cdk1	AGAAGGTACTTACGGTGTGGT	GAGAGATTTCCCGAATTGCAGT
Decr1	GATCCGGGTCCTCAGAGGTTT	ATCAGGTGGTAGCATAGGCTT
Dhfr	CGCTCAGGAACGAGTTCAAGT	TGCCAATTCCGGTTGTTCAATA
Fxn	CCACGCCCATTTGAACCTC	TCTTTCATACGCTGTCTCGTCT
Hnf4a	GGTAGGGGAGAATGCGACTC	AAACTCCAGGGTGGTGTAGG
Krt7	CACCCGGAATGAGATTGCG	GCACGCTGGTTCTTCAAGGT
Krt19	TGCTGGATGAGCTGACTCTG	AATCCACCTCCACACTGACC
Rcc1	ATGCCACCCAAGCGCATAG	CAAGCCTGGTTCTGTGTTGTG
RhoA	AGCTTGTGGTAAGACATGCTTG	GTGTCCCATAAAGCCAACTCTAC
Smad4	GACAGTGTCTGTGTGAATCC	TACTTGGCGGGTGTTGGATG
Tgfb1	TACGTCAGACATTCGGGAAGCA	AGGTAACGCCAGGAATTGTTGC
Tgfb2	GCCTTCGCCCTCTTTACATTGA	CGGAAGCTTCGGGATTTATGGT
Tgfb3	CCTGGCCCTGCTGAACTTG	TTGATGTGGCCGAAGTCCAAC
